# The influence of negative training set size on machine learning-based virtual screening

**DOI:** 10.1186/1758-2946-6-32

**Published:** 2014-06-11

**Authors:** Rafał Kurczab, Sabina Smusz, Andrzej J Bojarski

**Affiliations:** 1 Department of Medicinal Chemistry, Institute of Pharmacology, Polish Academy of Sciences, Smętna 12, 31-343 Kraków, Poland; 2 Faculty of Chemistry, Jagiellonian University, R. Ingardena 3, 30-060 Kraków, Poland

## Abstract

**Background:**

The paper presents a thorough analysis of the influence of the number of negative training examples on the performance of machine learning methods.

**Results:**

The impact of this rather neglected aspect of machine learning methods application was examined for sets containing a fixed number of positive and a varying number of negative examples randomly selected from the ZINC database. An increase in the ratio of positive to negative training instances was found to greatly influence most of the investigated evaluating parameters of ML methods in simulated virtual screening experiments. In a majority of cases, substantial increases in precision and MCC were observed in conjunction with some decreases in hit recall. The analysis of dynamics of those variations let us recommend an optimal composition of training data. The study was performed on several protein targets, 5 machine learning algorithms (SMO, Naïve Bayes, Ibk, J48 and Random Forest) and 2 types of molecular fingerprints (MACCS and CDK FP). The most effective classification was provided by the combination of CDK FP with SMO or Random Forest algorithms. The Naïve Bayes models appeared to be hardly sensitive to changes in the number of negative instances in the training set.

**Conclusions:**

In conclusion, the ratio of positive to negative training instances should be taken into account during the preparation of machine learning experiments, as it might significantly influence the performance of particular classifier. What is more, the optimization of negative training set size can be applied as a boosting-like approach in machine learning-based virtual screening.

## Background

Machine learning (ML) methods are widely used in the process of drug discovery to classify molecules as potentially active or inactive against a particular protein target. The vast majority of those methods require the preparation of a training set of compounds (supervised learning) that are used to develop a decision rule that can be then applied to sort a dataset of new molecules (the test set) among particular activity classes [1]. A number of studies have aimed to determine optimal learning parameters and examine their impact on classification effectiveness [2-5]. Interestingly, no extensive research that considers the influence of the ratio of active to inactive training examples on the efficiency of new active compounds recognition has been performed. The possible impact of negative training examples on the performance of ML models has only recently become the subject of research in the field of cheminformatics. Although, it should be emphasized that the construction of the training set might be the issue of the well-known problem of learning from imbalanced data as well. However, due to a large number of existing approaches in this field, their relevant examination was beyond the scope of this paper. Recently, we showed that the way of inactive set design significantly influences classification effectiveness, with the best results obtained for training sets with inactives randomly selected from the ZINC database [6]. Tests were conducted with six of the most frequently used approaches for selecting assumed inactive compounds: random and diverse selection from the ZINC database [7], the MDDR database [8] and libraries generated according to the DUD methodology [9]. All experiments were performed using 5 different protein targets, 3 different fingerprints for molecular representation and seven ML algorithms. Concurrently, Heikamp et al. [10] analyzed the effects of alternative sets of negative training data and different background databases on support vector machine (SVM) modeling and virtual screening (VS). The results showed a clear influence of negative training examples on SVM search efficiency, with the best performance achieved when SVM models were trained and screened on a dataset randomly chosen from ZINC (experimentally confirmed active and inactive compounds were selected from PubChem Confirmatory Bioassays [11]). The authors also touched the problem of positive to negative training examples ratio and noted that increased numbers of reference compounds generally can lead to improvement in the prediction abilities of SVM. The models were derived on the basis of differently composed training sets containing confirmed inactive molecules or compounds randomly selected from the ZINC database as negatives.

In this study, we delve into the influence of increasing the number of negative instances used for training (with a fixed set of actives) on ML methods performance. At first, ligands (from the MDDR database) of 3 proteins were studied in details, and next the analysis was extended by 12 other targets (active compounds fetched from ChEMBL [12]) to confirm the applicability of found conclusions. This is an extension of our previous study that focused on determining the optimal formula for providing the maximum possible yield from machine learning-based virtual screening, taking into account another very important aspect of designing VS experiments.

## Results and discussion

The question raised in this study was how the performance of machine learning-based virtual screening depends on the size of the dataset of negative training examples. To address this issue, we have first performed initial calculations for 3 different protein targets (5-HT_1A_, HIV-1 protease and matrix metalloproteinase) with actives fetched from the MDDR database. Next, to broaden the scope of the study and to verify the obtained results, the set of targets was extended by 12 proteins belonging to different classes (enzymes, membrane proteins, transcription factors, transporter) and compounds stored in the freely available ChEMBL database – confirmatory tests. Five machine learning algorithms (SMO, Naïve Bayes, Ibk, J48 and Random Forest) and 2 types of molecular fingerprints (MACCS and CDK FP) were applied to datasets of a fixed number of positive training instances and varied the number of negative examples to obtain different active to inactive ratios (from 0.5 to 20 with a step size of 0.5). In order to show the relative diversity of actives towards inactives randomly selected from the ZINC database, the matrices of Tanimoto coefficients were calculated (see Additional file 1: Figure S1). It revealed that for the great majority of active ligands, there were found inactive compounds from ZINC that were characterized by high similarity, therefore the classification task (discrimination between actives and inactives) was not trivial.

The selection of particular classification algorithms was dictated by their popularity in virtual screening experiments. The machine learning methods performance was assessed with the use of the following evaluating metrics – recall, precision, Matthews Correlation Coefficient (MCC), ROC curves and AUC.

The recall, precision and MCC are usually used to provide comprehensive assessments of imbalanced learning problems. On the other hand, the ROC curves, are very useful for providing a visual representation of the relative trade-offs between the true positives and false positives of classification in regard to data distributions. Albeit, in the case of highly skewed data sets, the ROC curves may provide an overly optimistic view of an algorithm’s performance. In such situations the PR curves can provide a more informative representation of performance assessment [13]. As recall, precision and MCC results give slightly different information and should be differently interpreted, they were described independently from ROC and AUC. Recall, precision and MCC values are generated only on the basis of the confusion matrix, whereas ROC and AUC take into account the value of predictive function – therefore they provide information about the expected proportion of positives ranked before a uniformly drawn random negative.

### Initial tests

#### General search performance

**Figure 1:**
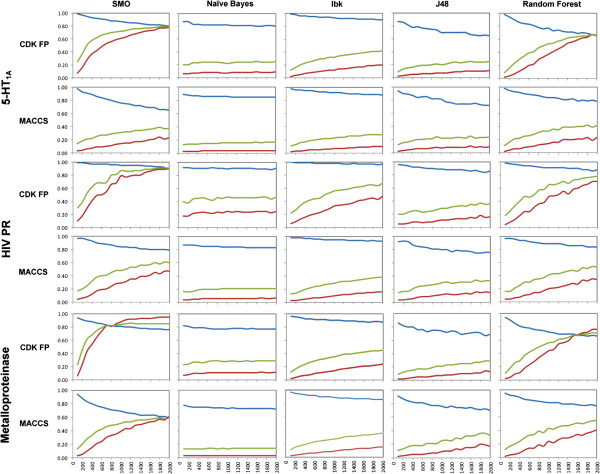
**The dependence of negative training set size on machine learning-based virtual screening performance for 2 types of fingerprints and 3 protein targets, averaged over 10 independent trials.** The colored lines denote the type of evaluated parameter used (blue – recall, red – precision and green – MCC). The figure presents the values of evaluating parameters (recall, precision and MCC) obtained in the experiments carried out for sets with fixed number of active molecules and varying number of inactives.

**Figure 2:**
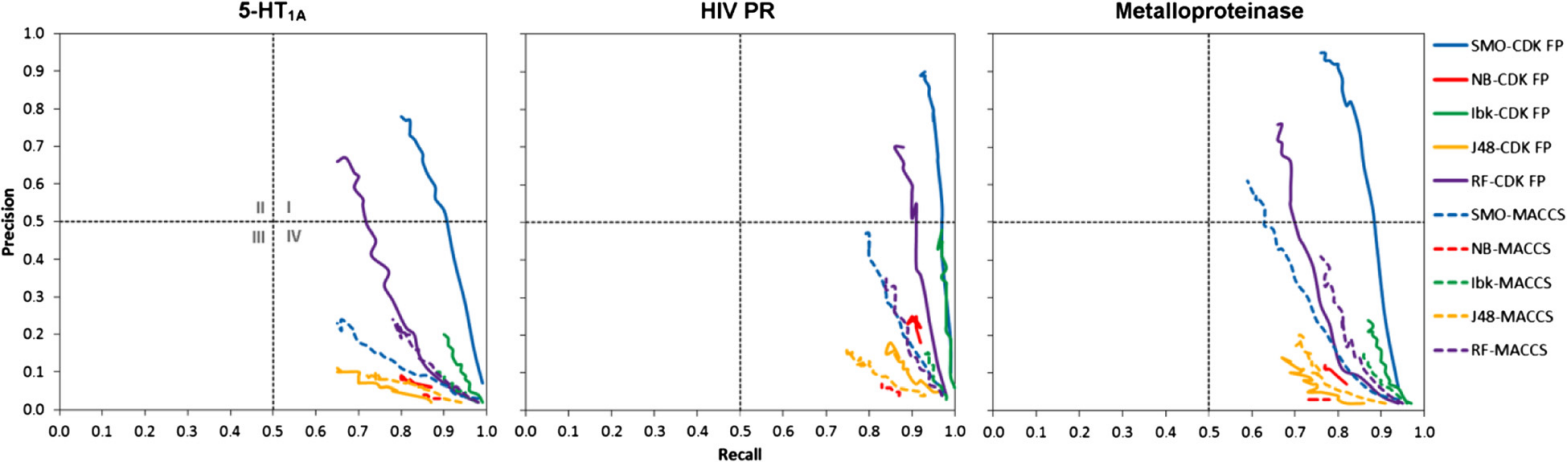
**Precision-recall plots illustrating the improvement of the machine learning models performance, for different fingerprints and protein targets induced by increasing the size of the negative training set.** The figure presents the changes in recall and precision values for different conditions of experiment according to the provided legend.

The recall, precision and MCC values for combinations of 3 initial targets with fingerprints and ML methods are presented in Figure 1. These results refer only to experiments with the maximum number of inactives equal to 2000, as further increase of the negative training size did not significantly change the evaluating parameters values (see Additional file 2: Figure S2, Additional file 3: Table S1, Additional file 4: Table S2). The single plot illustrates the relation between the averaged (after 10 iterations) value of a given performance measure and the negative set size for a particular combination of ML algorithm and molecular fingerprint.Global analysis showed that increasing the ratio of negative to positive training examples significantly improved the effectiveness of machine learning-based virtual screening. In each case, precision and MCC yielded the best results when the number of negatives was approximately in 9 to 10-fold excess to positives. Interestingly, the highest recall values (ranging from 0.8–1.0) were obtained when the number of positive samples was greater than the number of negative training samples (ratio approximately 1:0.5). In addition, analysis of precision-recall plots (Figure 2) showed that, initially, all models occupied the region of medium classification effectiveness with high recall and low precision (quarter IV). When the size of the negative set increased, performance improvements were observed for all methods except Naïve Bayes. The most significant changes were found for SMO and Random Forest methods, which both moved to the region of high recall and precision (quarter I).

**Figure 3:**
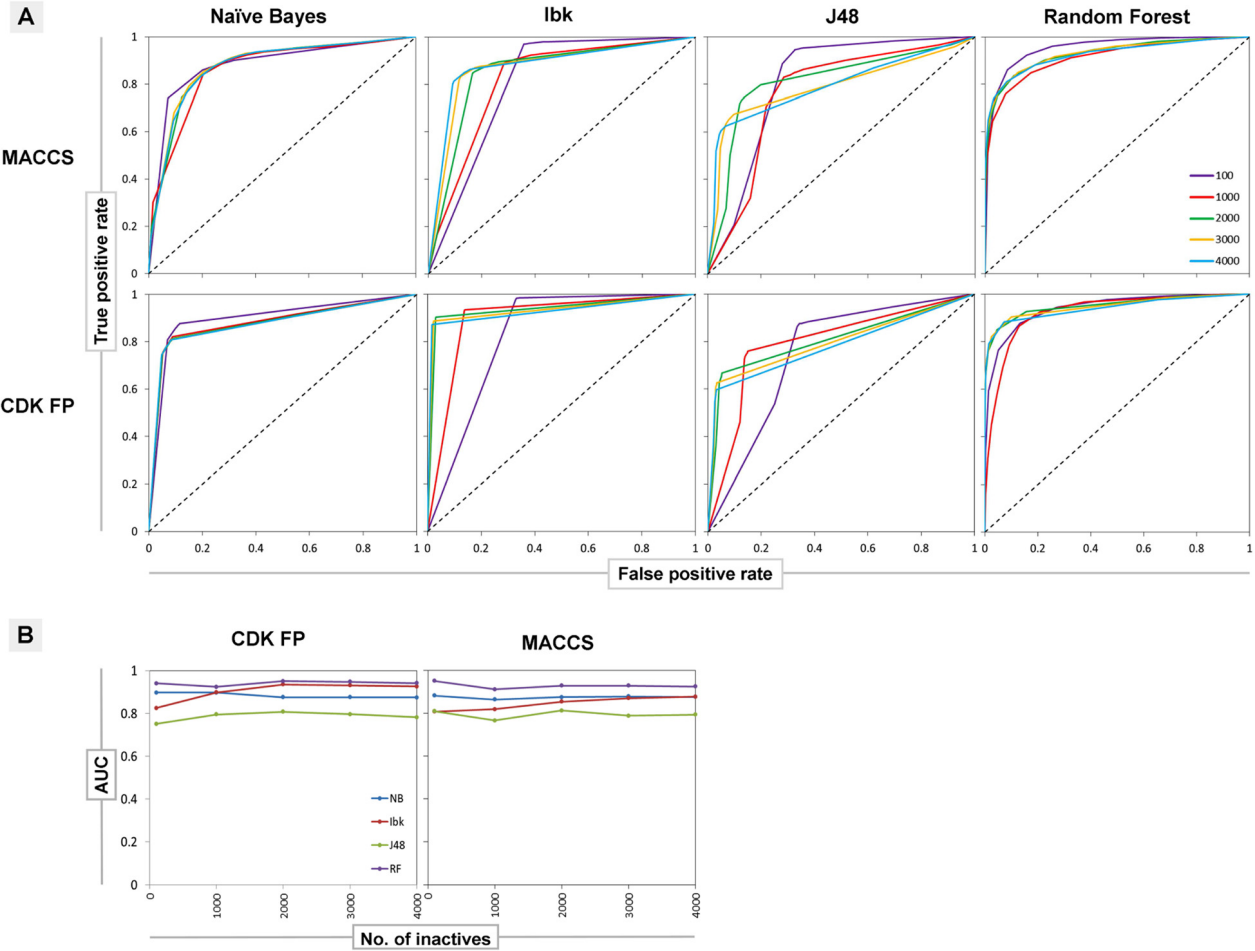
**The changes of the ROC curves and AUC values for different number of inactives used for ML training.** The figure presents ROC curves for 5-HT_1A_ target illustrating the improvement of the machine learning models performance, calculated for several negative training example quantities (i.e. 100, 1000, 2000, 3000 and 4000) **(A)** and the dependence of AUC on the number of inactive training examples **(B)**.

Interestingly, the analysis of ROC curves plotted for 5-HT_1A_ (Figure 3A) showed that increasing the ratio of inactives to actives, improved the ability of Ibk and J48 to correctly rank positive examples over the negative ones, whereas for Random Forest and Naïve Bayes this relationship is negligible. On the other hand, the AUC was the least affected parameter by the number of inactives used for training (Figure 3B). The described tendency was similar for all initial targets and fingerprints, and only slight differences were observed in the changes in the values of AUC. However, as it was mentioned previously, both ROC and AUC provide different type of information – that is not only classification itself, but they also take into account ranking, showing the rate of expectation that a uniformly drawn random positive is ranked before a uniformly drawn random negative. ROC and AUC analysis for HIV-1 protease and metalloproteinase are placed in the Additional file 5: Figure S3 and Additional file 6: Figure S4). The examination of ROC, AUC and other evaluating parameters, in the context of the way particular classification algorithm constructs the predictive model, is included in the Additional file 7.

#### Influence of negative training set size on the performance of ML methods

For almost all ML algorithms studied, increasing the number of negative training instances was found to decrease recall and increase both precision and MCC values. However, completely different tendencies were observed for the Naïve Bayes algorithm, which in all cases showed only a slight sensitivity to the negative training set size enlargement (Figure 2). According to its methodological assumptions it labels instances from the test set according to the class distribution from the training data. Therefore, one would expect that increasing the number of inactive compounds in the training set should lead to improvement of Naïve Bayes performance in virtual screening-like experiment. However, the attempts to reproduce the class distribution from the training set led to errors in class assignments for sets with higher number of inactives which in turn resulted in lower values of evaluating parameters instead of the expected uplift.Considering the dynamics of changes in ML performance with a growing number of negative training examples, SMO and Random Forest algorithms quickly led to models with very good classification effectiveness (Figure 2). In comparison, the improvement of Ibk and J48 methods was less significant; their corresponding curves on precision-recall plots responded very slowly to increases in the number of negative instances.

In general, the preferable ratio of active to inactive compounds in the training sets was found to be approximately 1:9–1:10 – further increase in the size of the negative training set led to only slight improvements in global ML methods performance (MCC) that were not profitable due to increases in computational expenses (see Additional file 2: Figure S2, Additional file 3: Table S1).

Table 1 shows the changes in given performance parameters for a particular ML method obtained between experiments with the lowest and the highest number of negative training examples. These quantitative results confirm that the largest improvement in precision and MCC values was observed for SMO (0.20–0.89 and 0.23–0.72, respectively) and Random Forest (0.22–0.74 and 0.31–0.60, respectively) methods. However, changes in recall were also observed – it was reduced even by 0.35 for the metalloproteinase when the number of training inactives was raised from 100 to 2000. Additionally, the Ibk algorithm produced the greatest decrease in recall and a modest improvement in precision (0.08–0.42) and MCC (0.17–0.46) when the size of the negative examples set was increased.

**Table 1:** The improvement of evaluating parameters calculated for targets from the initial tests

**ML/Fingerprint**		**5-HT**_**1A**_			**HIV PR**			**Metalloproteinase**		
		**R**	**P**	**MCC**	**R**	**P**	**MCC**	**R**	**P**	**MCC**
SMO	CDK FP	−0.19	0.71	0.54	−0.06	0.80	0.61	−0.18	0.89	0.72
	MACCS	−0.34	0.20	0.23	−0.18	0.43	0.43	−0.35	0.58	0.47
NB	CDK FP	−0.07	0.03	0.05	−0.01	0.07	0.07	−0.05	0.05	0.06
	MACCS	−0.04	0.01	0.04	−0.04	0.03	0.04	−0.06	0.00	0.01
Ibk	CDK FP	−0.09	0.18	0.30	−0.03	0.42	0.46	−0.09	0.22	0.33
	MACCS	−0.1	0.08	0.17	−0.06	0.13	0.26	−0.11	0.13	0.25
J48	CDK FP	−0.22	0.09	0.16	−0.20	0.11	0.16	−0.17	0.11	0.20
	MACCS	−0.22	0.07	0.12	−0.16	0.11	0.18	−0.21	0.15	0.22
RF	CDK FP	−0.34	0.64	0.56	−0.10	0.66	0.60	−0.28	0.74	0.60
	MACCS	−0.20	0.22	0.31	−0.13	0.30	0.37	−0.19	0.39	0.43

The table shows the changes in given performance parameters for a particular ML method obtained between experiments with the lowest and the highest ratio of negative to positive training examples.

#### Fingerprint dependency

From fingerprint-based point of view, the overall tendency of searching did not change considerably when MACCS or CDK FP fingerprints were used. In almost all cases, the total improvement of predictive models was much lower for MACCS fingerprints (Table 1). This is also shown through the precision-recall plots (Figure 2), where in almost all cases studied, the performance of a particular ML algorithm changed more dynamically when molecules were encoded by CDK FPs than MACCS fingerprints. Moreover, only in one case MACCS (in combination with SMO for metalloproteinase, Figure 2) produced effective classification models (quarter I). Interestingly, CDK FP required approximately 8-fold more time on training prediction models and database screening than MACCS. This is likely caused by the difference in the length of the bit string used to represent the molecule (166 and 1024 bit positions used in MACCS and CDK FP, respectively).

### Confirmatory tests

Machine learning experiments were also carried out in an analogous approach for compounds selected from the ChEMBL database. Different classes of proteins with various number of known active molecules provided covering of a broad range of tested cases. The results were analyzed in a similar way as it was in case of the previous part of the study (Table 2, Additional file 8: Figure S5, Additional file 9: Table S3). The outcome of these experiments is compatible with this from the initial tests – increasing the number of inactives included in the training data provides the substantial improvement of classification efficiency. Interestingly, the results are also consistent from the quantitative point of view – improvement in MCC values for SMO and Random Forest was 0.25–0.62 and 0.44–0.74, respectively). The recall, precision and MCC values obtained in confirmatory tests are visualized in Additional file 8: Figure S5. The results for particular algorithms are in line with those from the previous part and the global conclusions are the same: several fold excess of inactives in relation to actives constitutes an optimal solution for machine learning experiments – although further addition of inactives usually led to further increase in evaluating parameters values, the level of improvement was inadequate to the computational time needed.

**Table 2:** The improvement of MCC values calculated for targets from the confirmatory tests

**Target**	**ChEMBL class**	**Train/test**	**CDK FP/MCC**				
			**SMO**	**NB**	**Ibk**	**J48**	**RF**
D2	membrane receptor	310/1407	0.55	0.04	0.48	0.25	0.65
EGFR	enzyme/kinase	280/1303	0.35	0.09	0.50	0.41	0.61
Mu opioid	unclassified protein	270/1235	0.53	0.05	0.55	0.33	0.65
SERT	transporter	390/1822	0.25	0.03	0.62	0.40	0.55
Estrogen α	transcription factor	133/614	0.47	0.10	0.46	0.28	0.67
AChE	enzyme/hydrolase	162/743	0.58	0.10	0.41	0.23	0.69
Factor Xa	enzyme/protease	530/2439	0.54	0.02	0.58	0.39	0.67
Thrombin	enzyme/protease	370/1691	0.59	0.04	0.58	0.27	0.66
PDE5	enzyme/phosphodiesterase	152/695	0.56	0.01	0.36	0.28	0.60
Renin	enzyme/protease	340/1556	0.46	0.06	0.59	0.33	0.65
Glucocorticoid	transcription factor	236/1084	0.62	0.03	0.56	0.31	0.74
CRF1	membrane receptor	200/914	0.59	0.03	0.46	0.34	0.74

The table shows the changes in MCC for a particular ML method obtained between experiments with the lowest and the highest ratio of negative to positive training examples.

## Experimental

### Compound data sets

The MDDR database was used as a source of the structures of active compounds for 3 different protein targets: 5-HT_1A_R agonists, HIV-1 protease inhibitors and metalloproteinase inhibitors used in the initial study. Targets, belonging to diverse families of proteins and possessing large numbers of ligands, were carefully chosen after a survey of the literature concerning different aspects of ML methods tests [14-16].

The ML models were built and tested using active compounds from the MDDR database and assumed inactives randomly selected from ZINC v. 10 (details presented in Table 3). In each iteration step, the negative training set was rebuilt with a number of inactive compounds varying from 100 to 4000 in increments of 100. The positive training set was fixed and composed of approx. 18% of all compounds with confirmed activity toward the particular target. The remaining actives, together with the same number of 99000 compounds randomly selected from ZINC, formed the test set. For each ratio of active to inactive compounds, 10 trials were performed.

**Table 3:** Composition of training and test sets used in the initial study

**Protein target**	**MDDR activity index**	**Number of actives/inactives**	
		**Training set**	**Test set**
5-HT_1A_	06235	198/100–2000	903/99000
HIV PR	71523	203/100–2000	932/99000
Metalloproteinase	78432	144/100–2000	644/99000

The changes in recall, precision and MCC values between particular iterations were statistically insignificant, and therefore repeating the study with another randomly selected ZINC sets leads to very similar results and the dependencies connected with the number of inactives in the training set are preserved.

The ChEMBL Target Classification Hierarchy directed the selection of 12 targets used in the confirmatory tests, which ensured the diversity of both proteins and structures of active compounds. As ChEMBL (unlike MDDR) contains numerical values of particular parameter determining compounds activity, active compounds were selected manually by setting an appropriate threshold – only compounds which annotated activity satisfied one of the conditions: *K*_i_ < 100 nM or IC_50_ < 200 nM, were included into active class. Because different number of active ligands were obtained, the chosen number of inactives was rescaled to ensure the same active to inactive ratios, as in initial study. Details concerning number of actives in both training and test sets are included in Table 2.

### Machine learning algorithms

Five of the most commonly used in cheminformatics ML algorithms were selected: Sequential Minimal Optimization (SMO) [17], Naïve Bayes classifier (NB) [18], Instance-Based Learning (Ibk) [19,20], J48 [21] and Random Forest (RF) [22,23]. All machine learning calculations were carried out using the WEKA package (version 3.6).

### Calculations and performance measures

Compound structures were represented using MACCS structural keys [24] and CDK standard hashed fingerprints with a default path length of 6 (FP) [25], generated by PaDEL-Descriptor software [26].

To evaluate the virtual screening performance of ML methods, the following parameters were used (averaged over 10 trials): recall – R (1), precision – P (2), Mathews Correlation Coefficient – MCC (3) and receiver operating characteristic (ROC) and the area under the ROC curve (AUC).

(1) $$R=\frac{\mathrm{TP}}{\mathrm{TP}+\mathrm{FN}}$$

(2) $$P=\frac{\mathrm{TP}}{\mathrm{TP}+\mathrm{FP}}$$

(3) $$\mathrm{MCC}=\frac{\mathrm{TP}\cdot \mathrm{TN}-\mathrm{FP}\cdot \mathrm{FN}}{\sqrt{\left(\mathrm{TP}+\mathrm{FP}\right)\cdot \left(\mathrm{TP}+\mathrm{FN}\right)\cdot \left(\mathrm{TN}+\mathrm{FP}\right)\cdot \left(\mathrm{TN}+\mathrm{FN}\right)}}$$

Recall measures the number of correctly identified positive instances, precision describes the correctness of positive predictions and the MCC is a balanced measure of binary classification effectiveness, ranging from −1 to 1, with 1 referring to perfect prediction. The Receiver Operator Characteristic curves (ROC) present how the number of correctly classified positive examples varies with the number of incorrectly predicted negative examples. Unfortunately, it was not possible to obtain ROC curve and AUC for SMO algorithm, because used WEKA implementation enables only on the binary classification.

These parameters were selected to enable the assessment of classification effectiveness from various perspectives. All calculations were performed on an Intel Core i7 CPU 3.00 GHz computer system with 24 GB RAM running a 64-bit Linux operating system.

## Conclusions

We have investigated a seldom-explored question in machine learning-based virtual screening methodology: how the performance of machine learning depends on the size of the set of negative training examples. We compared a variety of combinations of machine learning algorithms in classification experiments using compounds represented by 2 types of molecular fingerprints, for sets generated on the basis of confirmed active (from 2 activity databases) and varied numbers of assumed inactive compounds randomly selected from ZINC.

We found that the ratio of positive to negative training instances should be taken into account during the preparation of machine learning experiments, as it might significantly influence the performance of particular classifier. In general, increasing the size of the negative training set (with a constant quantity of positives), led to decrease in recall and improvement in precision, whereas no significant effect on AUC values was observed. However, the precision changes were much higher than the changes in recall, and hence the global classification effectiveness expressed by MCC values was enhanced by the addition of more inactives to the training data. These findings are generally in line with the results obtained by Heikamp et al. [10], who found better performance for an increased number of negative training examples for SVM models. An exception was the Naïve Bayes algorithm, for which no significant changes in models’ performance were observed. This provides some evidence of its independence of training set perturbation and variation. Overall, the best models (in terms of MCC values) were obtained for combination of SMO with CDK FP. The results were consistent for all protein targets and fingerprints, however, fingerprint with shorter bit string (MACCS) demonstrated less ability to improve ML models. For all the analyzed scenarios (target/ML method/fingerprint) and sizes of used test set, the preferable ratio of positive to negative training instances was found to be approximately 1:9 to 1:10. These findings revealed that the optimization of negative training set size can be applied as a boosting-like approach in machine learning-based virtual screening. However, it should be noted that the preferable active:inactive ratio indicated in the study might change under different experimental conditions (the dimension of the screening database, different number of actives, relative diversity of actives towards inactives, used to train the ML algorithms, application of methods for imbalanced learning, optimization of ML methods parameters, etc.), but this require further research which goes far beyond the scope of the article.

## Methods

The machine learning methods used in the study are gathered in Table 4. The default settings of all the tested classifiers were applied.

**Table 4:** Machine learning algorithms used and a short description of their training parameters

**Classifier**	**Classification scheme**	**Settings**
Sequential Minimal Optimization (SMO)	functions	The complexity parameter was set at 1, the epsilon for a round-off error was 1.0 E-12, and the option of normalizing training data was chosen. The normalized polynomial kernel was used.
Naïve Bayes (NB)	bayes	–
Instance-Based Learning (Ibk)	lazy	The nearest neighbor search algorithm using the Euclidean distance function and 1 neighbor.
J48	trees	C.4.5 pruning
Random Forest (RF)	trees	Trees with unlimited depth, seed number: 1. Number of generated trees: 10.

## Competing interests

The authors declare that they have no competing interests.

## Authors’ contributions

All authors designed the experiments. SS and RK performed the experiments. All authors analyzed the data and draw conclusions and wrote, read and approved the final manuscript.

## Supplementary Material

Additional file 1: Figure S1 The diversity analysis of compounds used in experiments in terms of Tanimoto coefficient calculated for each pair of structures in the dataset. The figure presents plots illustrating the dependence of Tanimoto coefficients calculated between all positive train examples and randomly selected negative training examples. Click here for file

Additional file 2: Figure S2 The dependence of negative training set size on machine learning-based virtual screening performance, calculated for 5-HT_1A_ over the full range of 100–4000 negative examples. The figure shows values of the evaluating parameters (recall, precision, MCC) for the extended range of the number of inactives present in the training set, that is 100–4000 for experiments for 5-HT_1A_. Click here for file

Additional file 3: Table S1 The changes in performance parameters calculated as the difference between average values obtained for number of negative training examples varying from 2100–4000. Changes in the parameters calculated for 100–2000 inactives are shown in parentheses. The table compares the magnitude of changes for the number of inactives varying from 100–2000 and 2100–4000. It is clearly visible that increasing the number of inactives over 2000 did not affect so much the ML algorithms performance as it was in case of the lower number on inactive compounds used for training. Click here for file

Additional file 4: Table S2 Detailed results of the calculations for 5-HT_1A_. The table presents the numerical values of all evaluating parameters obtained for the experiments with 5-HT_1A_ ligands. Click here for file

Additional file 5: Figure S3 ROC analysis for HIV-1 protease and metalloproteinase. The figure presents ROC curves for HIV-1 protease and metalloproteinase. Click here for file

Additional file 6: Figure S4 AUC analysis for HIV-1 protease and metalloproteinase. The figure presents AUC curves for HIV-1 protease and metalloproteinase. Click here for file

Additional file 7 **Additional comments on machine learning algorithms and the dependency of their results on the number of inactives in the training data.** The file contains the additional comments on the machine learning algorithms used in the study, relating the obtained results with the theories lying behind each classification model. Click here for file

Additional file 8: Figure S5 The dependence of negative training set size on machine learning-based virtual screening performance for 2 types of fingerprints and 12 protein targets from confirmatory set, averaged over 10 independent trials. The colored lines denote the type of evaluated parameter used (blue – recall, red – precision and green – MCC). The figure visualizes the evaluating parameters (recall, precision, MCC) values obtained for fixed number of actives and varied number of inactives in the confirmatory experiments stage. Click here for file

Additional file 9: Table S3 Changes in performance parameters calculated as the differences between average values obtained for the lowest and highest ratio of negative to positive training examples obtained for all ChEMBL targets. The table shows the changes in given performance parameters for a particular ML method obtained between experiments with the lowest and the highest number of negative training examples for protein targets selected for confirmatory tests. Click here for file
