# Supplementary material for: The influence of negative training set size on machine learning-based virtual screening
Source: J Cheminform. 2014 Jun 11;6:32. doi: 10.1186/1758-2946-6-32 (PMC4061540; doi:10.1186/1758-2946-6-32)

Figure S1. The diversity analysis of compounds used in experiments in terms of Tanimoto coefficient calculated for each pair of structures in the dataset. Blue circles represent maximum value of this parameter, red – minimum one, and green – the average value over the whole set.

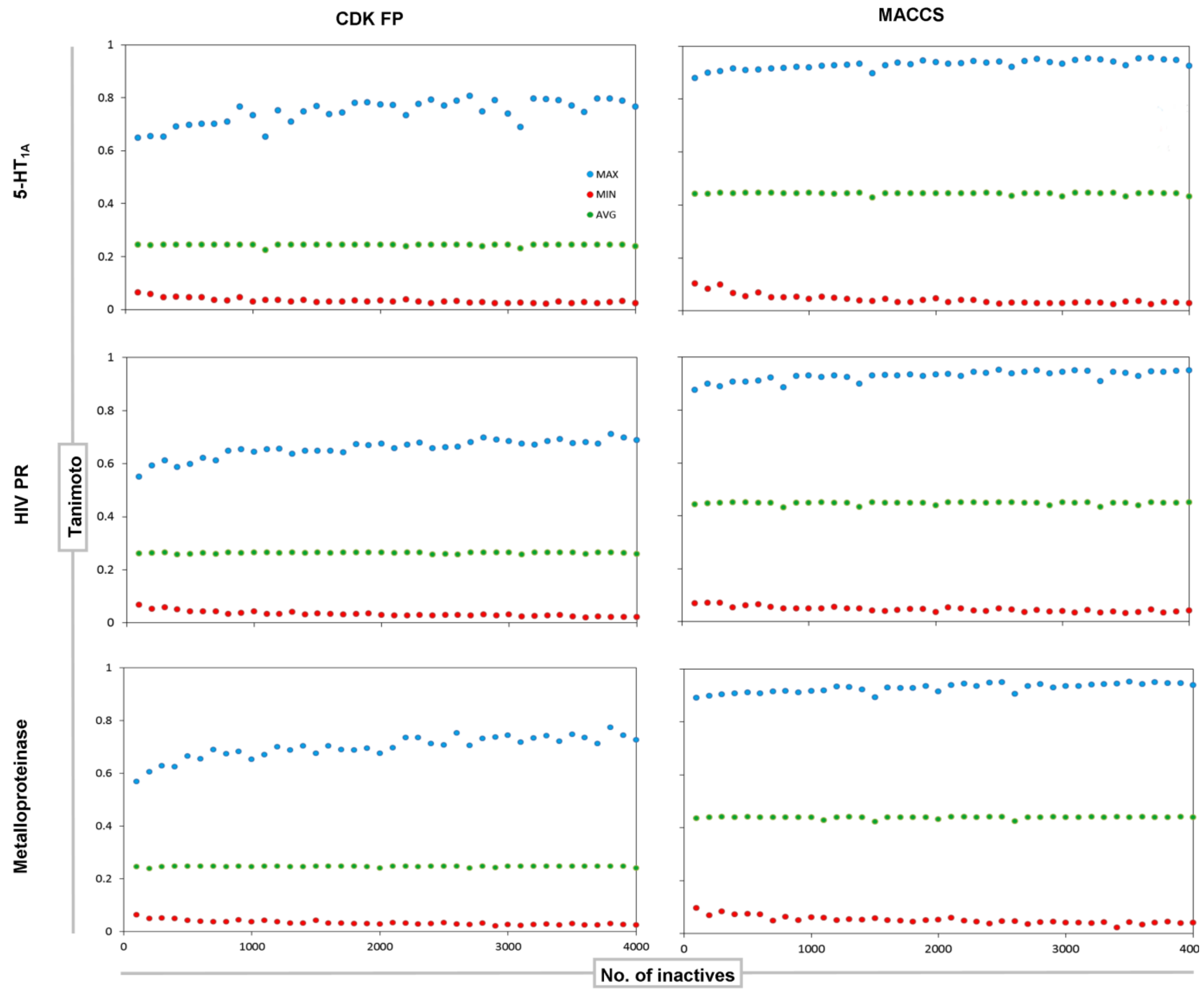

Supplement: Additional file 1: Figure S1 — The diversity analysis of compounds used in experiments in terms of Tanimoto coefficient calculated for each pair of structures in the dataset. The figure presents plots illustrating the dependence of Tanimoto coefficients calculated between all positive train examples and randomly selected negative training examples. [file 1758-2946-6-32-S1.pdf]
