# Supplementary material for: The influence of negative training set size on machine learning-based virtual screening
Source: J Cheminform. 2014 Jun 11;6:32. doi: 10.1186/1758-2946-6-32 (PMC4061540; doi:10.1186/1758-2946-6-32)

Figure S2. The dependence of negative training set size on machine learning-based virtual screening performance, calculated for 5-HT_{1A} over the full range of 100–4000 negative examples.

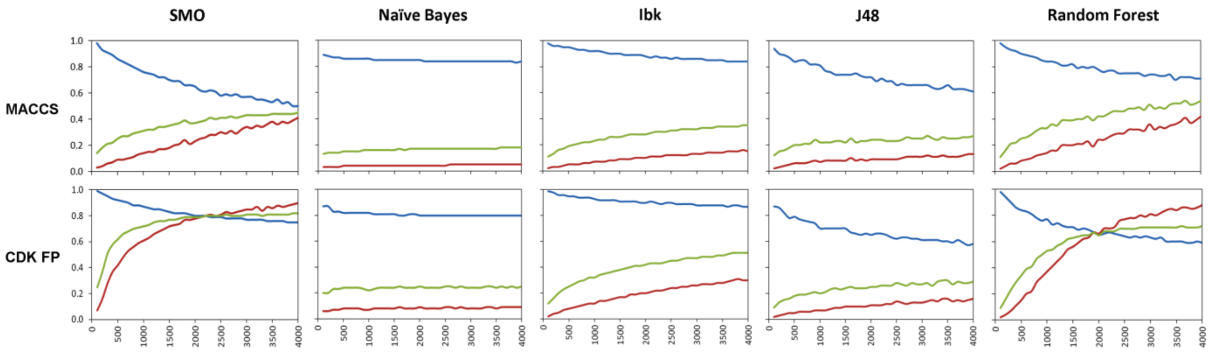

Supplement: Additional file 2: Figure S2 — The dependence of negative training set size on machine learning-based virtual screening performance, calculated for 5-HT1A over the full range of 100–4000 negative examples. The figure shows values of the evaluating parameters (recall, precision, MCC) for the extended range of the number of inactives present in the training set, that is 100–4000 for experiments for 5-HT1A. [file 1758-2946-6-32-S2.pdf]
