# Supplementary material for: The influence of negative training set size on machine learning-based virtual screening
Source: J Cheminform. 2014 Jun 11;6:32. doi: 10.1186/1758-2946-6-32 (PMC4061540; doi:10.1186/1758-2946-6-32)
Supplement: Additional file 3: Table S1 — The changes in performance parameters calculated as the difference between average values obtained for number of negative training examples varying from 2100–4000. Changes in the parameters calculated for 100–2000 inactives are shown in parentheses. The table compares the magnitude of changes for the number of inactives varying from 100–2000 and 2100–4000. It is clearly visible that increasing the number of inactives over 2000 did not affect so much the ML algorithms performance as it was in case of the lower number on inactive compounds used for training. [file 1758-2946-6-32-S3.pdf]

Table S1. The changes in performance parameters calculated as the difference between average values obtained for number of negative training examples varying from 2100–4000. Changes in the parameters calculated for 100–2000 inactives are shown in parentheses.

ML/Fingerprint		5-HT_{1A}		
		Recall	Precision	MCC
SMO	CDK FP	-0.05 (-0.19)	0.11 (0.71)	0.13 (0.54)
	MACCS	-0.12 (-0.34)	0.16 (0.20)	0.07 (0.23)
NB	CDK FP	-0.07 (-0.07)	0.01 (0.03)	0.01 (0.05)
	MACCS	0.00 (-0.04)	0.01 (0.01)	0.01 (0.04)
Ibk	CDK FP	-0.04 (-0.09)	0.09 (0.18)	0.08 (0.30)
	MACCS	-0.03 (-0.1)	0.04 (0.08)	0.06 (0.17)
J48	CDK FP	-0.08 (-0.22)	0.05 (0.09)	0.04 (0.16)
	MACCS	-0.08 (-0.22)	0.04 (0.07)	0.03 (0.12)
RF	CDK FP	-0.07 (-0.34)	0.18 (0.64)	0.04 (0.56)
	MACCS	-0.05 (-0.20)	0.18 (0.22)	0.12 (0.31)
