# Supplementary material for: The influence of negative training set size on machine learning-based virtual screening
Source: J Cheminform. 2014 Jun 11;6:32. doi: 10.1186/1758-2946-6-32 (PMC4061540; doi:10.1186/1758-2946-6-32)
Supplement: Additional file 4: Table S2 — Detailed results of the calculations for 5-HT1A. The table presents the numerical values of all evaluating parameters obtained for the experiments with 5-HT1A ligands. [file 1758-2946-6-32-S4.pdf]

Table S2. Detailed results of the calculations for 5-HT$_{1A}$.

Negative set	SMO - MACCS			SMO – CDK FP		
	Recall	Precision	MCC	Recall	Precision	MCC
100	0.98	0.03	0.14	0.99	0.07	0.25
200	0.93	0.04	0.18	0.97	0.16	0.37
300	0.91	0.06	0.21	0.95	0.28	0.51
400	0.89	0.07	0.22	0.93	0.37	0.58
500	0.86	0.09	0.25	0.92	0.42	0.62
600	0.84	0.09	0.27	0.91	0.48	0.66
700	0.82	0.1	0.27	0.9	0.53	0.68
800	0.8	0.11	0.29	0.88	0.56	0.7
900	0.78	0.13	0.3	0.88	0.59	0.71
1000	0.76	0.14	0.31	0.87	0.61	0.72
1100	0.75	0.15	0.32	0.86	0.63	0.73
1200	0.74	0.15	0.32	0.85	0.66	0.75
1300	0.72	0.17	0.34	0.85	0.68	0.76
1400	0.72	0.17	0.34	0.84	0.70	0.76
1500	0.7	0.18	0.35	0.83	0.72	0.77
1600	0.69	0.2	0.36	0.82	0.73	0.77
1700	0.69	0.21	0.37	0.82	0.74	0.78
1800	0.66	0.24	0.39	0.82	0.77	0.79
1900	0.66	0.21	0.37	0.81	0.77	0.79
2000	0.65	0.23	0.37	0.8	0.78	0.79
2100	0.62	0.25	0.38	0.8	0.79	0.79
2200	0.61	0.26	0.39	0.8	0.80	0.80
2300	0.62	0.28	0.41	0.79	0.81	0.8
2400	0.61	0.28	0.4	0.79	0.8	0.8
2500	0.58	0.3	0.41	0.79	0.81	0.8
2600	0.59	0.29	0.41	0.78	0.83	0.81
2700	0.58	0.31	0.42	0.78	0.82	0.8
2800	0.59	0.29	0.41	0.78	0.83	0.8
2900	0.57	0.32	0.42	0.78	0.84	0.8
3000	0.57	0.34	0.43	0.77	0.85	0.81
3100	0.57	0.33	0.43	0.77	0.85	0.81
3200	0.55	0.35	0.43	0.77	0.87	0.81
3300	0.55	0.34	0.43	0.77	0.84	0.8
3400	0.54	0.36	0.43	0.76	0.87	0.81
3500	0.53	0.38	0.44	0.76	0.86	0.81
3600	0.55	0.36	0.44	0.76	0.88	0.81
3700	0.52	0.38	0.44	0.76	0.87	0.81
3800	0.53	0.37	0.44	0.75	0.88	0.81
3900	0.5	0.39	0.44	0.75	0.89	0.82
4000	0.5	0.41	0.45	0.75	0.9	0.82

Negative set	Naïve Bayes - MACCS			Naïve Bayes – CDK FP		
	Recall	Precision	MCC	Recall	Precision	MCC
100	0.89	0.03	0.13	0.87	0.06	0.20
200	0.88	0.03	0.14	0.87	0.06	0.20
300	0.87	0.03	0.14	0.83	0.07	0.23
400	0.87	0.03	0.14	0.83	0.07	0.23
500	0.86	0.04	0.15	0.82	0.08	0.24
600	0.86	0.04	0.15	0.82	0.08	0.24
700	0.86	0.04	0.15	0.82	0.08	0.24
800	0.86	0.04	0.15	0.82	0.08	0.24
900	0.86	0.04	0.16	0.82	0.07	0.23
1000	0.86	0.04	0.16	0.82	0.07	0.22
1100	0.85	0.04	0.16	0.81	0.08	0.23
1200	0.85	0.04	0.16	0.81	0.08	0.24
1300	0.85	0.04	0.16	0.81	0.08	0.24
1400	0.85	0.04	0.16	0.81	0.08	0.24
1500	0.85	0.04	0.16	0.81	0.08	0.24
1600	0.85	0.04	0.17	0.8	0.09	0.25
1700	0.85	0.04	0.16	0.8	0.08	0.24
1800	0.85	0.04	0.17	0.81	0.08	0.24
1900	0.85	0.04	0.17	0.81	0.08	0.24
2000	0.85	0.04	0.17	0.8	0.09	0.25
2100	0.84	0.04	0.17	0.8	0.08	0.24
2200	0.84	0.04	0.17	0.8	0.08	0.24
2300	0.84	0.04	0.17	0.8	0.08	0.24
2400	0.84	0.04	0.17	0.8	0.08	0.24
2500	0.84	0.04	0.17	0.8	0.09	0.25
2600	0.84	0.05	0.17	0.8	0.09	0.25
2700	0.84	0.05	0.17	0.8	0.08	0.24
2800	0.84	0.05	0.17	0.8	0.09	0.25
2900	0.84	0.05	0.17	0.8	0.08	0.24
3000	0.84	0.05	0.17	0.8	0.08	0.24
3100	0.84	0.05	0.17	0.8	0.08	0.24
3200	0.84	0.05	0.17	0.8	0.08	0.24
3300	0.84	0.05	0.17	0.8	0.09	0.25
3400	0.84	0.05	0.17	0.8	0.09	0.25
3500	0.84	0.05	0.17	0.8	0.08	0.24
3600	0.84	0.05	0.18	0.8	0.09	0.25
3700	0.84	0.05	0.18	0.8	0.09	0.24
3800	0.84	0.05	0.18	0.8	0.09	0.25
3900	0.83	0.05	0.18	0.8	0.09	0.24
4000	0.84	0.05	0.18	0.8	0.09	0.25

Negative set	Ibk - MACCS			Ibk – CDK FP		
	Recall	Precision	MCC	Recall	Precision	MCC
100	0.98	0.02	0.11	0.99	0.02	0.12

200	0.96	0.03	0.13	0.98	0.04	0.16
300	0.96	0.03	0.16	0.96	0.05	0.2
400	0.95	0.04	0.17	0.96	0.07	0.23
500	0.95	0.05	0.19	0.95	0.08	0.25
600	0.94	0.05	0.2	0.95	0.09	0.27
700	0.93	0.05	0.21	0.94	0.1	0.29
800	0.93	0.06	0.22	0.94	0.11	0.3
900	0.92	0.06	0.22	0.94	0.12	0.32
1000	0.92	0.07	0.23	0.93	0.12	0.32
1100	0.92	0.07	0.24	0.92	0.14	0.34
1200	0.91	0.07	0.24	0.92	0.14	0.35
1300	0.90	0.08	0.26	0.92	0.15	0.36
1400	0.90	0.08	0.26	0.92	0.16	0.37
1500	0.9	0.09	0.26	0.91	0.17	0.38
1600	0.89	0.09	0.27	0.91	0.17	0.39
1700	0.89	0.09	0.28	0.91	0.19	0.4
1800	0.89	0.1	0.28	0.91	0.19	0.41
1900	0.89	0.1	0.28	0.9	0.2	0.41
2000	0.88	0.1	0.28	0.9	0.2	0.42
2100	0.98	0.02	0.11	0.99	0.02	0.12
2200	0.96	0.03	0.13	0.98	0.04	0.16
2300	0.96	0.03	0.16	0.96	0.05	0.2
2400	0.95	0.04	0.17	0.96	0.07	0.23
2500	0.95	0.05	0.19	0.95	0.08	0.25
2600	0.94	0.05	0.2	0.95	0.09	0.27
2700	0.93	0.05	0.21	0.94	0.1	0.29
2800	0.93	0.06	0.22	0.94	0.11	0.3
2900	0.92	0.06	0.22	0.94	0.12	0.32
3000	0.92	0.07	0.23	0.93	0.12	0.32
3100	0.92	0.07	0.24	0.92	0.14	0.34
3200	0.91	0.07	0.24	0.92	0.14	0.35
3300	0.90	0.08	0.26	0.92	0.15	0.36
3400	0.90	0.08	0.26	0.92	0.16	0.37
3500	0.9	0.09	0.26	0.91	0.17	0.38
3600	0.89	0.09	0.27	0.91	0.17	0.39
3700	0.89	0.09	0.28	0.91	0.19	0.4
3800	0.89	0.1	0.28	0.91	0.19	0.41
3900	0.89	0.1	0.28	0.9	0.2	0.41
4000	0.88	0.1	0.28	0.9	0.2	0.42

Negative set	J48 - MACCS			J48 – CDK FP		
	Recall	Precision	MCC	Recall	Precision	MCC
100	0.94	0.02	0.12	0.87	0.02	0.09
200	0.90	0.03	0.15	0.86	0.03	0.13
300	0.89	0.04	0.16	0.82	0.04	0.15
400	0.87	0.05	0.18	0.78	0.05	0.16

500	0.84	0.06	0.2	0.79	0.05	0.18
600	0.85	0.06	0.2	0.77	0.06	0.19
700	0.85	0.06	0.21	0.76	0.06	0.19
800	0.82	0.07	0.21	0.75	0.06	0.2
900	0.82	0.08	0.24	0.74	0.07	0.21
1000	0.81	0.07	0.22	0.7	0.07	0.2
1100	0.77	0.08	0.22	0.70	0.07	0.21
1200	0.76	0.08	0.22	0.7	0.08	0.23
1300	0.74	0.08	0.23	0.7	0.09	0.24
1400	0.74	0.08	0.23	0.70	0.09	0.23
1500	0.74	0.08	0.22	0.7	0.1	0.24
1600	0.74	0.1	0.25	0.67	0.1	0.25
1700	0.75	0.08	0.22	0.66	0.1	0.24
1800	0.73	0.09	0.23	0.65	0.1	0.24
1900	0.72	0.08	0.23	0.66	0.1	0.24
2000	0.72	0.09	0.24	0.65	0.11	0.25
2100	0.94	0.02	0.12	0.87	0.02	0.09
2200	0.90	0.03	0.15	0.86	0.03	0.13
2300	0.89	0.04	0.16	0.82	0.04	0.15
2400	0.87	0.05	0.18	0.78	0.05	0.16
2500	0.84	0.06	0.2	0.79	0.05	0.18
2600	0.85	0.06	0.2	0.77	0.06	0.19
2700	0.85	0.06	0.21	0.76	0.06	0.19
2800	0.82	0.07	0.21	0.75	0.06	0.2
2900	0.82	0.08	0.24	0.74	0.07	0.21
3000	0.81	0.07	0.22	0.7	0.07	0.2
3100	0.77	0.08	0.22	0.70	0.07	0.21
3200	0.76	0.08	0.22	0.7	0.08	0.23
3300	0.74	0.08	0.23	0.7	0.09	0.24
3400	0.74	0.08	0.23	0.70	0.09	0.23
3500	0.74	0.08	0.22	0.7	0.1	0.24
3600	0.74	0.1	0.25	0.67	0.1	0.25
3700	0.75	0.08	0.22	0.66	0.1	0.24
3800	0.73	0.09	0.23	0.65	0.1	0.24
3900	0.72	0.08	0.23	0.66	0.1	0.24
4000	0.72	0.09	0.24	0.65	0.11	0.25

Negative set	**Random Forest - MACCS**			**Random Forest – CDK FP**		
	Recall	Precision	MCC	Recall	Precision	MCC
100	0.98	0.02	0.11	0.98	0.02	0.09
200	0.95	0.04	0.16	0.94	0.04	0.16
300	0.93	0.06	0.21	0.9	0.07	0.23
400	0.92	0.06	0.22	0.86	0.11	0.29
500	0.9	0.08	0.25	0.84	0.15	0.34
600	0.89	0.09	0.26	0.83	0.2	0.39
700	0.88	0.1	0.28	0.81	0.22	0.41

800	0.87	0.12	0.31	0.78	0.29	0.47
900	0.85	0.13	0.33	0.76	0.33	0.5
1000	0.84	0.14	0.33	0.77	0.37	0.53
1100	0.84	0.16	0.36	0.73	0.41	0.54
1200	0.83	0.16	0.35	0.74	0.45	0.57
1300	0.81	0.20	0.39	0.72	0.49	0.59
1400	0.81	0.20	0.39	0.71	0.54	0.62
1500	0.82	0.2	0.39	0.71	0.56	0.63
1600	0.79	0.21	0.4	0.69	0.59	0.64
1700	0.8	0.21	0.4	0.7	0.62	0.65
1800	0.79	0.23	0.42	0.69	0.63	0.65
1900	0.8	0.19	0.39	0.67	0.67	0.67
2000	0.78	0.24	0.42	0.65	0.66	0.65
2100	0.98	0.02	0.11	0.98	0.02	0.09
2200	0.95	0.04	0.16	0.94	0.04	0.16
2300	0.93	0.06	0.21	0.9	0.07	0.23
2400	0.92	0.06	0.22	0.86	0.11	0.29
2500	0.9	0.08	0.25	0.84	0.15	0.34
2600	0.89	0.09	0.26	0.83	0.2	0.39
2700	0.88	0.1	0.28	0.81	0.22	0.41
2800	0.87	0.12	0.31	0.78	0.29	0.47
2900	0.85	0.13	0.33	0.76	0.33	0.5
3000	0.84	0.14	0.33	0.77	0.37	0.53
3100	0.84	0.16	0.36	0.73	0.41	0.54
3200	0.83	0.16	0.35	0.74	0.45	0.57
3300	0.81	0.20	0.39	0.72	0.49	0.59
3400	0.81	0.20	0.39	0.71	0.54	0.62
3500	0.82	0.2	0.39	0.71	0.56	0.63
3600	0.79	0.21	0.4	0.69	0.59	0.64
3700	0.8	0.21	0.4	0.7	0.62	0.65
3800	0.79	0.23	0.42	0.69	0.63	0.65
3900	0.8	0.19	0.39	0.67	0.67	0.67
4000	0.78	0.24	0.42	0.65	0.66	0.65
