# Supplementary figures and images for: The influence of negative training set size on machine learning-based virtual screening
Source: J Cheminform. 2014 Jun 11;6:32. doi: 10.1186/1758-2946-6-32 (PMC4061540; doi:10.1186/1758-2946-6-32)

**Figure S3.** ROC analysis for HIV-1 protease and metalloproteinase.

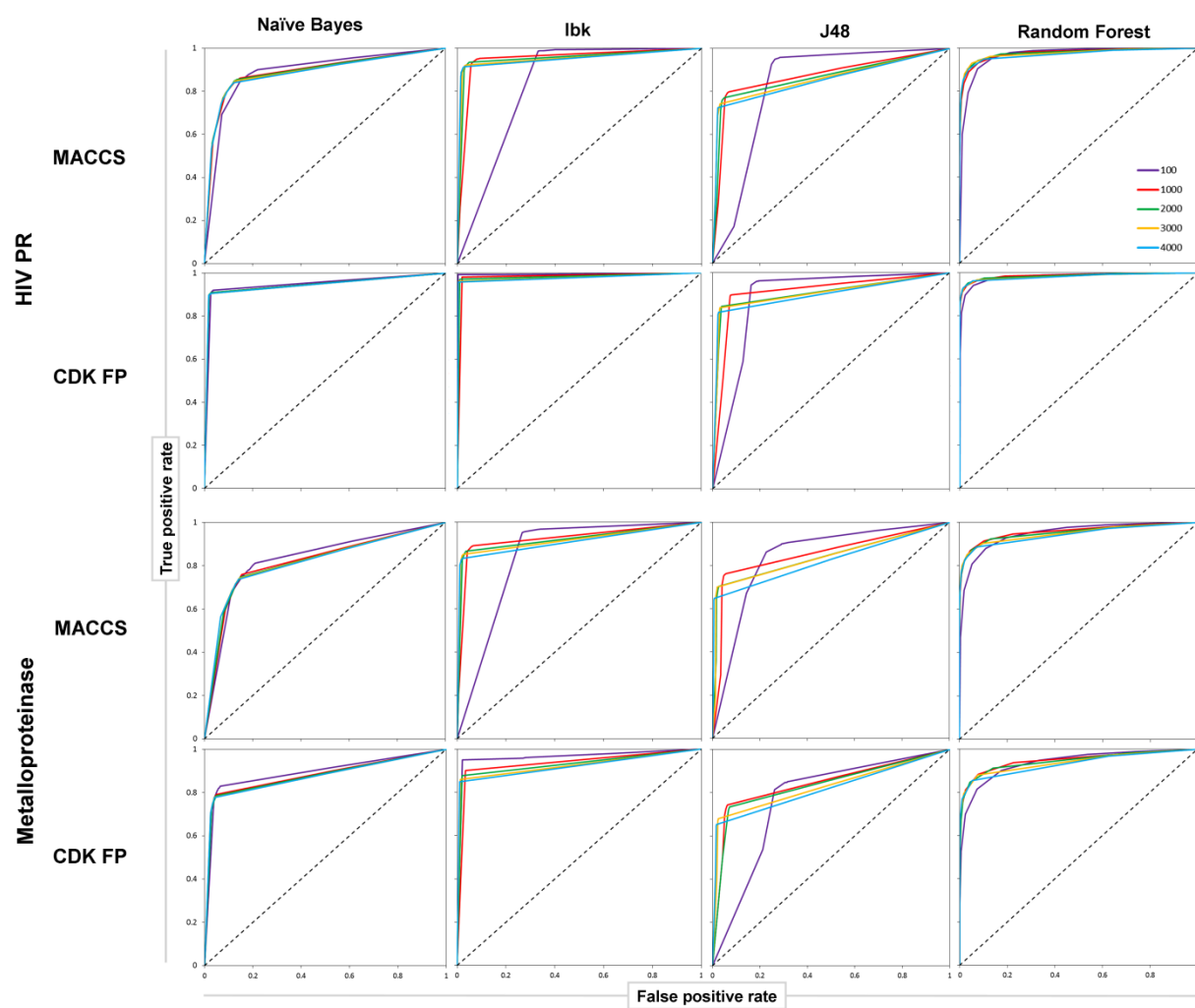

Supplement: Additional file 5: Figure S3 — ROC analysis for HIV-1 protease and metalloproteinase. The figure presents ROC curves for HIV-1 protease and metalloproteinase. [file 1758-2946-6-32-S5.pdf]

**Figure S4.** AUC analysis for HIV-1 protease and metalloproteinase.

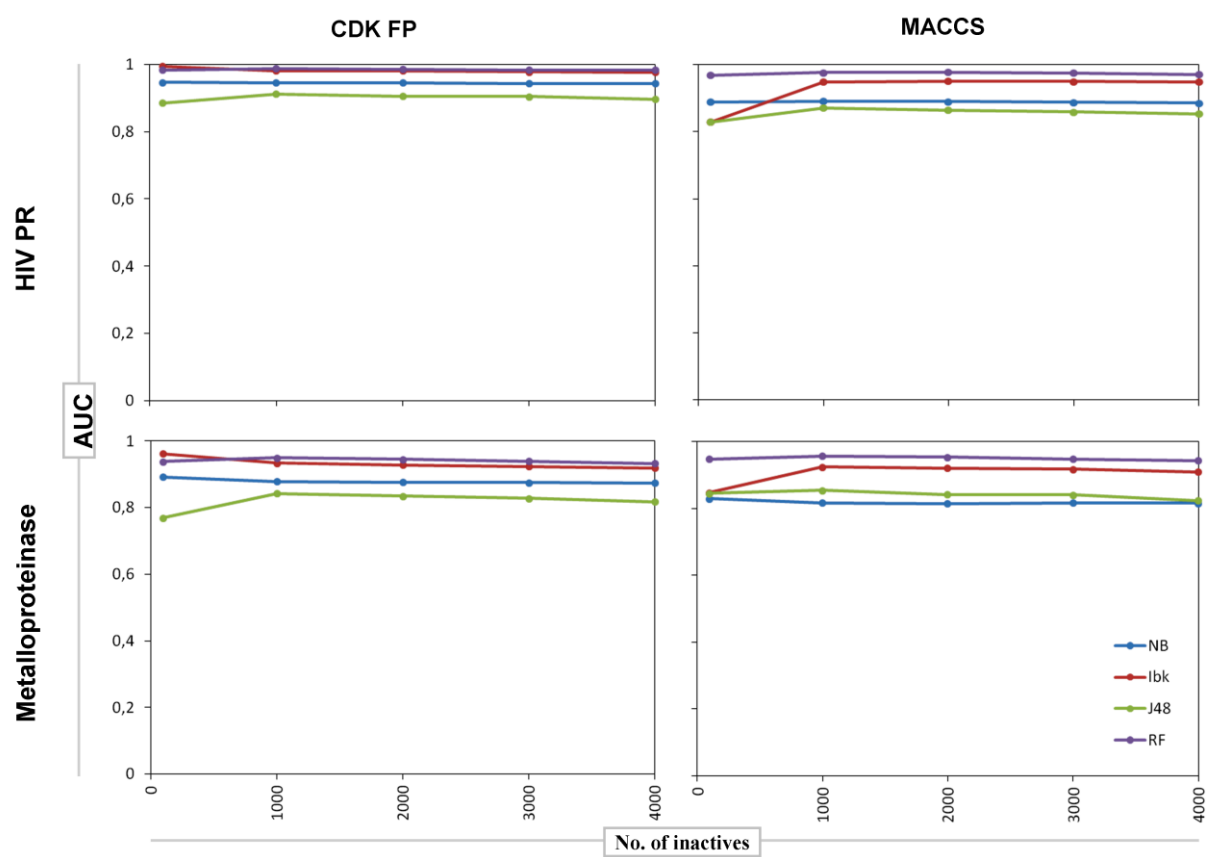

Supplement: Additional file 6: Figure S4 — AUC analysis for HIV-1 protease and metalloproteinase. The figure presents AUC curves for HIV-1 protease and metalloproteinase. [file 1758-2946-6-32-S6.pdf]
