# Supplementary material for: The influence of negative training set size on machine learning-based virtual screening
Source: J Cheminform. 2014 Jun 11;6:32. doi: 10.1186/1758-2946-6-32 (PMC4061540; doi:10.1186/1758-2946-6-32)
Supplement: Additional file 7 — Additional comments on machine learning algorithms and the dependency of their results on the number of inactives in the training data. The file contains the additional comments on the machine learning algorithms used in the study, relating the obtained results with the theories lying behind each classification model. [file 1758-2946-6-32-S7.pdf]

Discussion of used machine learning algorithms

Naïve Bayes algorithm is based on the Bayes theorem and the assumption that particular attributes are conditionally independent. Obviously, this assumption in usually not true (therefore a classifier is called "naïve"). The algorithm calculates the probability of belonging to particular class independently; as the final answer, the class with the highest probability is selected. Therefore, the ROC curves for Naïve Bayes could provide slightly different information than it is in case of the other classifiers. Labeling the compounds as active or inactive is not based on the absolute value of probability of belonging to particular class but simply the class with the highest probability is chosen. That is why for example in one case, probability of being active equals to 0.6 is sufficient for a given compound to label it as active (if the probability of being inactive is lower), and there could also be cases that having the probability of 0.9, the compound is inactive (if the probability of being active was lower, e.g. 0.89).

Ibk is very simple classifier, using k-nearest neighbour algorithm. The predictive model is not constructed in this case – for each of the analyzed compounds its distance (Euclidean distance) from each structure from the dataset is being calculated and the compound is assigned into the class in which there is present an example with the lowest distance to particular protein. Therefore, in this case, ROC curves are constructed on the basis of these distances from particular example in the training set. As a result, the scale is reversed, as intuitively lower distances from particular examples should mean the most reliable predictions (in 'normal' case, the higher probability of belonging to particular class, the better).

Random Forest and J48 are representatives of decision trees. They are constructed in a way that in each node there is placed an attribute that provides the best discrimination between actives and inactives on particular level. In case of Random Forest, a multitude of decision trees is being constructed during training and the final outcome is a result of voting for particular class. Consideration of this multiple answers is visible in ROC curves as the dependency on the number of inactives is much more visible in case of J48 than Random Forest although the performance evaluated in terms of recall, precision and MCC is much more diversified in case of Random Forest. This relationship for ROC curves is a result of voting – the final predictions are more consistent, although the final class assignment is not always correct and depends quite strongly on the number of inactives in the training set.

SMO is an algorithm solving the quadratic programming (QP) problem that arises during the training of support vector machines (SVM). The task of SVM is to construct a hyperplane or set of hyperplanes, which can be used for classification. If examples are not linearly separable, the transformation of original data with the use of kernel function is required. In case of SMO, the dependency of the results on the number of inactives in the training data is connected with the fact, that we used unweighted form of this algorithm and therefore, the more inactives in the training data, the model was more focused on proper classification of inactive compounds in order to maximize the accuracy of the predictions.

That is why a systematic growth of MCC with the increasing number of inactives is observed for this model.
