# Supplementary material for: The influence of negative training set size on machine learning-based virtual screening
Source: J Cheminform. 2014 Jun 11;6:32. doi: 10.1186/1758-2946-6-32 (PMC4061540; doi:10.1186/1758-2946-6-32)
Supplement: Additional file 8: Figure S5 — The dependence of negative training set size on machine learning-based virtual screening performance for 2 types of fingerprints and 12 protein targets from confirmatory set, averaged over 10 independent trials. The colored lines denote the type of evaluated parameter used (blue – recall, red – precision and green – MCC). The figure visualizes the evaluating parameters (recall, precision, MCC) values obtained for fixed number of actives and varied number of inactives in the confirmatory experiments stage. [file 1758-2946-6-32-S8.pdf]

Figure S5. The dependence of negative training set size on machine learning-based virtual screening performance for two types of fingerprints and twelve protein targets from confirmatory set, averaged over 10 independent trials. The colored lines denote the type of evaluated parameter used (blue – recall, red – precision and green – MCC).

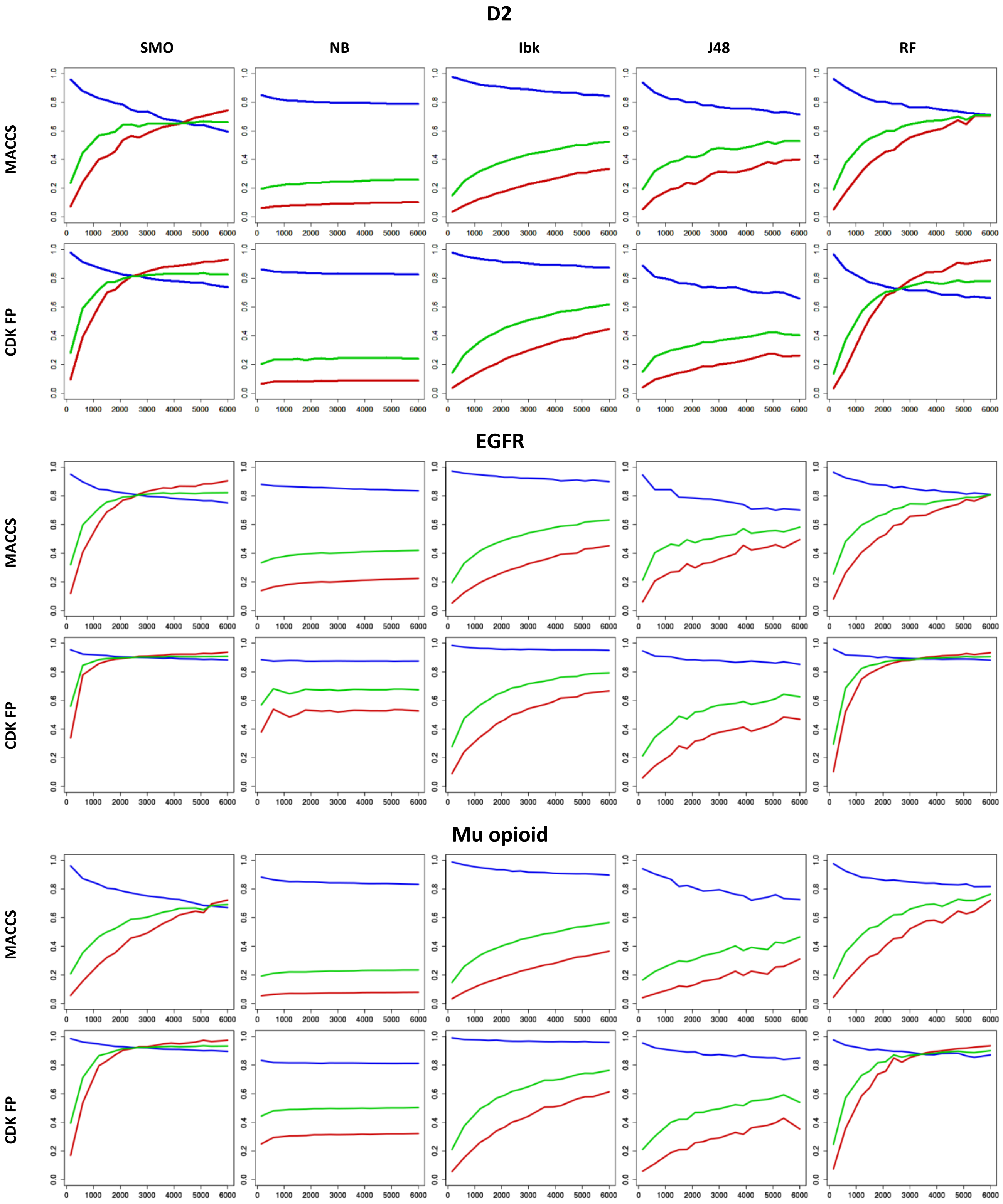

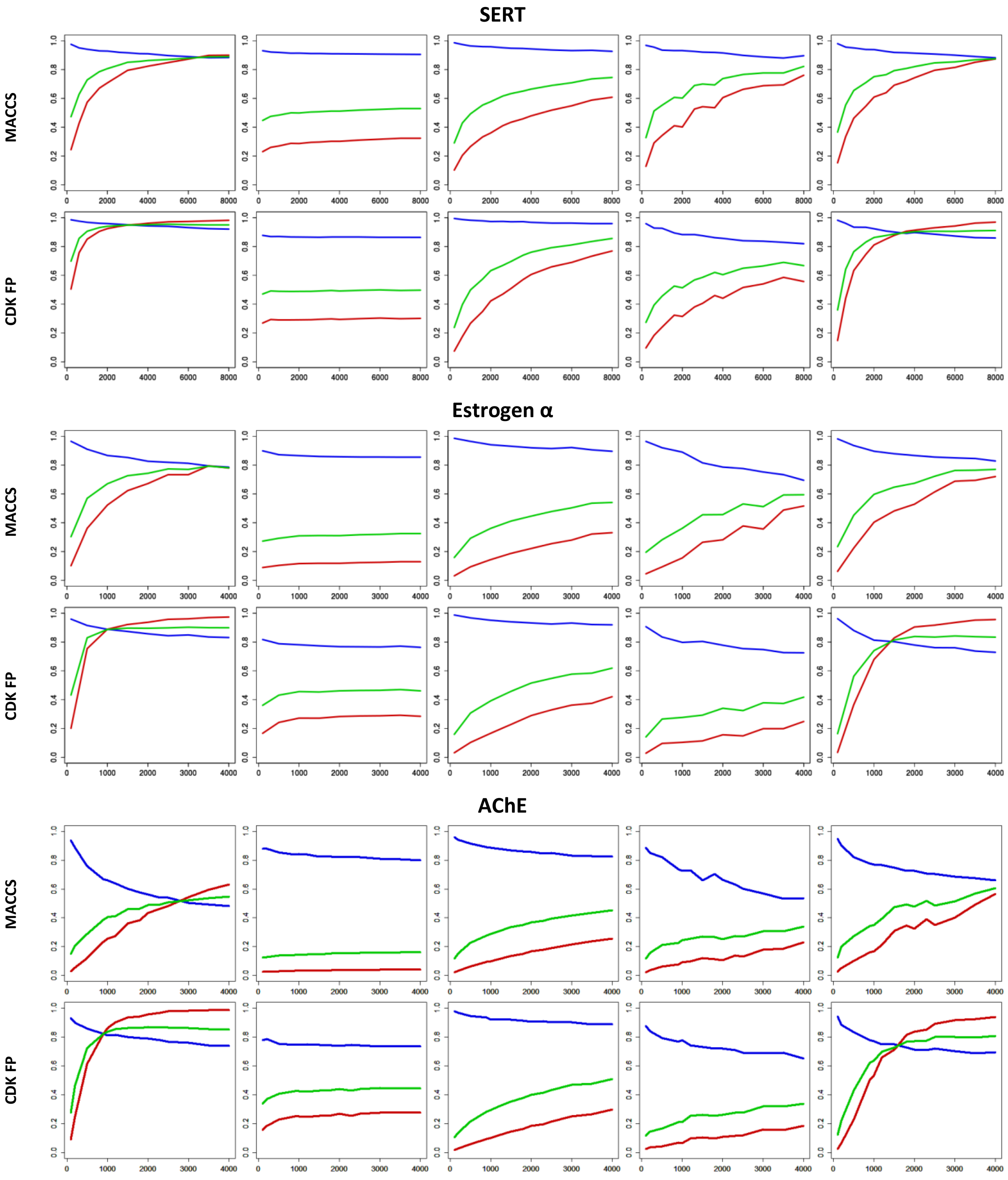
SERT
MACCS
CDK FP
Estrogen α
MACCS
CDK FP
AChE
MACCS
CDK FP

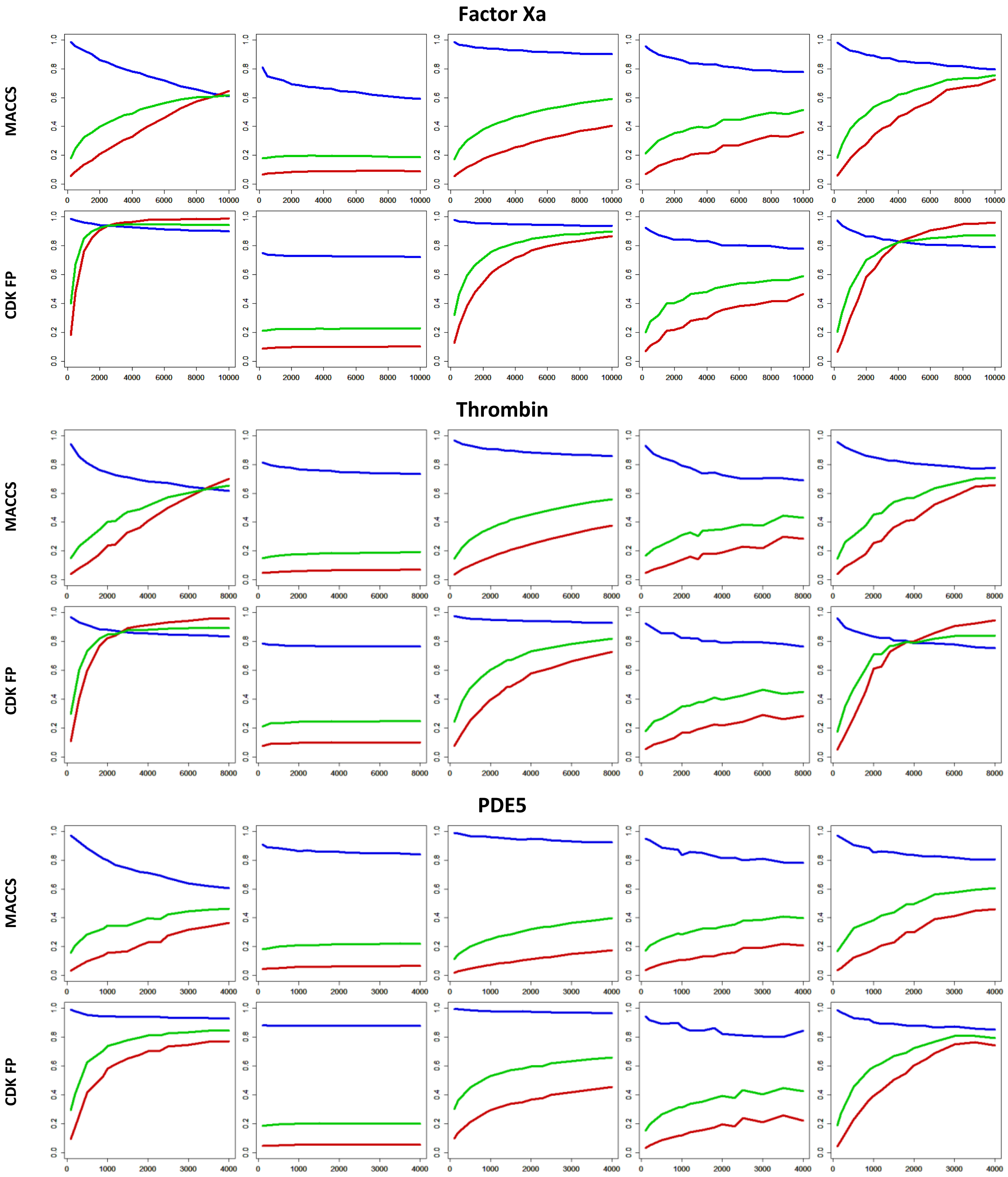
Factor Xa
MACCS
CDK FP
Thrombin
MACCS
CDK FP
PDE5
MACCS
CDK FP

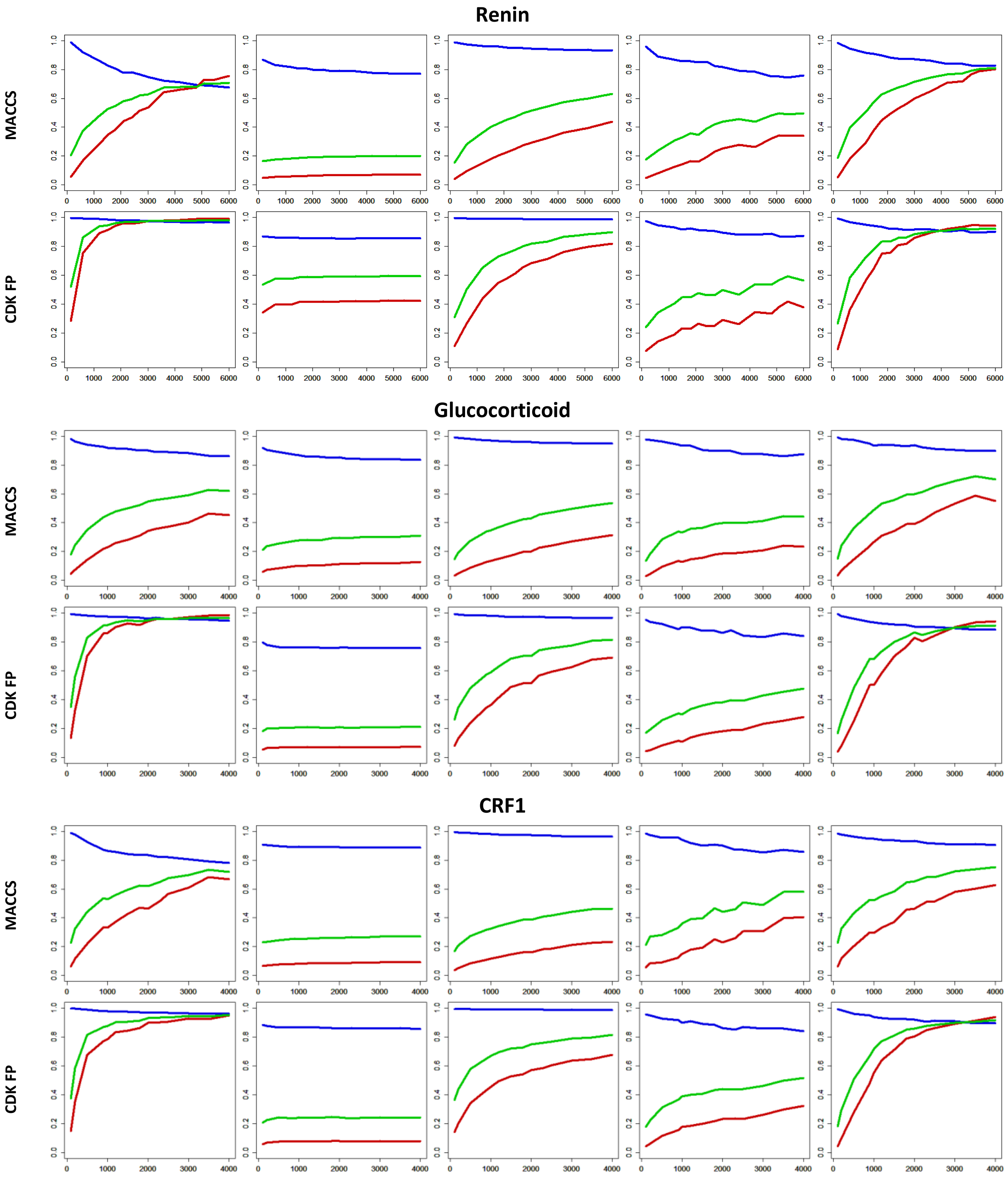
Renin
MACCS
CDK FP
Glucocorticoid
MACCS
CDK FP
CRF1
MACCS
CDK FP
