# Supplementary material for: The influence of negative training set size on machine learning-based virtual screening
Source: J Cheminform. 2014 Jun 11;6:32. doi: 10.1186/1758-2946-6-32 (PMC4061540; doi:10.1186/1758-2946-6-32)
Supplement: Additional file 9: Table S3 — Changes in performance parameters calculated as the differences between average values obtained for the lowest and highest ratio of negative to positive training examples obtained for all ChEMBL targets. The table shows the changes in given performance parameters for a particular ML method obtained between experiments with the lowest and the highest number of negative training examples for protein targets selected for confirmatory tests. [file 1758-2946-6-32-S9.pdf]

Table S3. Changes in performance parameters calculated as the differences between average values obtained for the lowest and highest ratio of negative to positive training examples obtained for selected ChEMBL targets.

Target/ Fingerprint		SMO			NB			Ibk			J48			RF		
		R	P	MCC	R	P	MCC	R	P	MCC	R	P	MCC	R	P	MCC
**D2**	CDK FP	-0.24	0.84	0.55	-0.04	0.02	0.04	-0.11	0.41	0.48	-0.23	0.22	0.25	-0.30	0.90	0.65
	MACCS	-0.37	0.67	0.42	-0.06	0.04	0.06	-0.13	0.30	0.38	-0.22	0.35	0.33	-0.25	0.66	0.51
**EGFR**	CDK FP	-0.07	0.60	0.35	-0.01	0.15	0.10	-0.03	0.58	0.51	-0.09	0.41	0.41	-0.08	0.83	0.61
	MACCS	-0.20	0.78	0.50	-0.04	0.08	0.09	-0.07	0.40	0.44	-0.24	0.43	0.37	-0.15	0.73	0.55
**Mu opioid**	CDK FP	-0.09	0.80	0.54	-0.02	0.07	0.06	-0.03	0.56	0.55	-0.10	0.29	0.33	-0.10	0.86	0.65
	MACCS	-0.29	0.67	0.48	-0.05	0.03	0.04	-0.09	0.33	0.42	-0.21	0.27	0.30	-0.16	0.68	0.59
**SERT**	CDK FP	-0.06	0.48	0.25	-0.01	0.03	0.03	-0.04	0.69	0.62	-0.14	0.46	0.39	-0.12	0.82	0.55
	MACCS	-0.09	0.66	0.42	-0.03	0.09	0.08	-0.06	0.51	0.45	-0.07	0.63	0.49	-0.10	0.72	0.51
**Estrogen α**	CDK FP	-0.13	0.77	0.47	-0.05	0.12	0.10	-0.07	0.39	0.46	-0.18	0.22	0.28	-0.23	0.92	0.67
	MACCS	-0.18	0.68	0.48	-0.04	0.04	0.05	-0.09	0.30	0.38	-0.27	0.47	0.40	-0.15	0.66	0.54
**AChE**	CDK FP	-0.20	0.90	0.58	-0.04	0.12	0.10	-0.09	0.28	0.41	-0.23	0.16	0.22	-0.25	0.91	0.68
	MACCS	-0.46	0.60	0.40	-0.08	0.02	0.04	-0.14	0.23	0.34	-0.35	0.21	0.22	-0.29	0.54	0.48
**Factor Xa**	CDK FP	-0.09	0.81	0.54	-0.03	0.01	0.02	-0.04	0.74	0.58	-0.15	0.40	0.39	-0.19	0.90	0.67
	MACCS	-0.38	0.59	0.44	-0.22	0.02	0.01	-0.08	0.35	0.42	-0.18	0.29	0.30	-0.18	0.67	0.57
**Thrombin**	CDK FP	-0.13	0.85	0.59	-0.02	0.24	0.04	-0.05	0.65	0.58	-0.16	0.23	0.27	-0.21	0.90	0.67
	MACCS	-0.32	0.66	0.50	-0.08	0.02	0.04	-0.11	0.34	0.41	-0.24	0.24	0.26	-0.18	0.62	0.56
**PDE5**	CDK FP	-0.06	0.68	0.55	0.00	0.01	0.01	-0.03	0.36	0.36	-0.10	0.19	0.27	-0.14	0.70	0.60
	MACCS	-0.37	0.33	0.31	-0.07	0.02	0.04	-0.07	0.16	0.28	-0.17	0.17	0.22	-0.16	0.42	0.44
**Renin**	CDK FP	-0.03	0.71	0.46	-0.01	0.08	0.06	-0.01	0.71	0.59	-0.10	0.30	0.33	-0.09	0.86	0.66
	MACCS	-0.32	0.70	0.51	-0.10	0.02	0.04	-0.05	0.40	0.48	-0.20	0.29	0.32	-0.16	0.75	0.63
**Glucocorticoid**	CDK FP	-0.05	0.85	0.62	-0.04	0.02	0.03	-0.03	0.61	0.55	-0.11	0.24	0.31	-0.11	0.91	0.75
	MACCS	-0.12	0.41	0.44	-0.08	0.07	0.10	-0.05	0.28	0.39	-0.10	0.20	0.31	-0.09	0.52	0.55
**CRF1**	CDK FP	-0.04	0.80	0.58	-0.03	0.02	0.04	-0.01	0.54	0.45	-0.12	0.28	0.34	-0.10	0.90	0.74
	MACCS	-0.21	0.61	0.49	-0.02	0.02	0.04	-0.03	0.20	0.30	-0.13	0.35	0.37	-0.08	0.57	0.52
